# PM2.5暴露通过诱导肿瘤相关巨噬细胞M2极化及IL-1α的分泌促进肺腺癌进展的机制研究

**DOI:** 10.3779/j.issn.1009-3419.2025.102.33

**Published:** 2025-09-20

**Authors:** Bomiao QING, Xiaolan LI, Qin RAN, Guoping LI

**Affiliations:** ^1^610031 成都，西南交通大学医学院; ^1^College of Medicine, Southwest Jiaotong University, Chengdu 610031, China; ^2^610031 成都，成都市第三人民医院呼吸健康研究所过敏与精准医学实验室; ^2^Laboratory of Allergy and Precision Medicine, Chengdu Institute of Respiratory Health, The Third People 's Hospital of Chengdu, Chengdu 610031, China

**Keywords:** 肺肿瘤, PM2.5, 肿瘤相关巨噬细胞, IL-1α, 肿瘤微环境, Lung neoplasms, PM2.5, Tumor-associated macrophages, Interleukin-1α, Tumor microenvironment

## Abstract

**背景与目的:**

肺腺癌（lung adenocarcinoma, LUAD）是全球发病率和死亡率均居前列的恶性肿瘤，其发生发展与肿瘤免疫微环境密切相关。近年来，大量研究表明，环境暴露因素在LUAD发生中具有重要作用，其中细颗粒物（PM2.5）作为空气污染的主要成分之一，已被证实与肺癌发病风险增加及预后不良密切相关。然而，PM2.5如何通过调控肿瘤免疫微环境促进LUAD进展的分子机制尚不清楚。肿瘤相关巨噬细胞（tumor-associated macrophages, TAMs），尤其是M2型巨噬细胞，在肿瘤生长、血管生成及免疫逃逸中起关键作用。本研究旨在探讨PM2.5暴露是否通过重塑TAMs功能促进LUAD进展，并筛选关键促癌因子。

**方法:**

本研究首先构建PM2.5暴露的小鼠原位肺癌模型，采用活体成像技术和流式细胞术分析肺部肿瘤生长及巨噬细胞表型变化。其次，通过巨噬细胞与肿瘤细胞共接种构建皮下移植瘤模型，进一步验证PM2.5对TAMs功能及肿瘤恶性行为的影响。结合体外细胞实验，运用流式细胞术、Western blot、逆转录定量聚合酶链反应（reverse transcription quantitative polymerase chain reaction, RT-qPCR）、细胞增殖试验（cell counting kit-8, CCK-8）、克隆形成及划痕实验，评估PM2.5对骨髓来源巨噬细胞（bone marrow-derived macrophage, BMDMs）表型及肿瘤细胞增殖、迁移与克隆形成能力的调控作用。最后，通过转录组测序并结合TISIDB（Tumor-immune System Interactions Database）、GEPIA（Gene Expression Profiling Interactive Analysis）等公共数据库进行生物信息学分析，筛选关键细胞因子并进行功能验证。

**结果:**

小鼠原位肺癌模型显示，PM2.5暴露可显著促进肺癌生长，并增加M2型TAMs比例（*P*<0.05）。皮下移植瘤模型显示，共接种PM2.5处理的BMDMs可明显促进肿瘤细胞增殖，且瘤内M2型TAMs比例上调。PM2.5预处理的BMDMs呈现程序性细胞死亡配体1（programmed cell death ligand 1, PD-L1）^+^、精氨酸酶1（arginase 1, ARG1）^+^免疫抑制表型，其条件培养基显著增强Lewis肺癌细胞（Lewis lung carcinoma, LLC）和黑色素瘤细胞（B16 melanoma cells, B16）迁移与克隆形成能力（*P*<0.05）。转录组测序结果显示，PM2.5处理显著改变巨噬细胞基因表达谱，其中白细胞介素-1α（interleukin-1α, IL-1α）作为关键上调的分泌因子，富集于免疫抑制相关信号通路。临床数据库分析进一步表明，IL-1α表达水平与LUAD免疫微环境中巨噬细胞及调节性T淋巴细胞（regulatory T cells, Treg）浸润程度呈正相关，且IL-1α高表达与LUAD患者较差的总生存期显著关联（HR=1.5, *P*=0.0053）。Western blot、RT-qPCR及免疫荧光实验证实，PM2.5暴露可显著上调TAMs中IL-1α的表达并促进其分泌。

**结论:**

PM2.5暴露通过诱导巨噬细胞获得免疫抑制表型并增强肿瘤细胞恶性行为，从而促进LUAD进展。机制上，IL-1α可能是PM2.5暴露环境下巨噬细胞分泌的关键促癌因子。本研究为PM2.5相关LUAD的发病机制提供了新见解，并提示IL-1α可能成为潜在治疗靶点。

## Abstract (en)

**Background and objective:**

Lung adenocarcinoma (LUAD) remains one of the leading causes of cancer morbidity and mortality worldwide, and its initiation and progression are closely associated with the tumor immune microenvironment. Increasing evidence suggests that environmental exposure is a critical factor influencing lung cancer development. Among these factors, fine particulate matter (PM2.5), a major component of air pollution, has been strongly linked to elevated lung cancer risk and unfavorable prognosis. However, the underlying immunoregulatory mechanisms by which PM2.5 drives LUAD progression remain poorly understood. Tumor-associated macrophages (TAMs), especially those polarized toward the M2 phenotype, are key components of the tumor microenvironment and play crucial roles in tumor growth, angiogenesis, and immune evasion. This study aims to investigate the effects of PM2.5 exposure on TAMs and to identify the key pro-tumorigenic factors mediating this process.

**Methods:**

A mouse orthotopic lung cancer model under PM2.5 exposure was established to assess lung tumor growth and macrophage phenotypic alterations using *in vivo* imaging and flow cytometry. A subcutaneous tumor model involving co-inoculated macrophages and tumor cells was used to further verify the effects of PM2.5 on the function of TAMs and tumor malignancy. Combining *in vitro* experiments, flow cytometry, Western blot, reverse transcription quantitative polymerase chain reaction (RT-qPCR), cell counting kit-8 (CCK-8) assay, colony formation assay, and wound healing assay were employed to evaluate the regulatory effects of PM2.5 on the polarization of bone marrow-derived macrophages (BMDMs) as well as tumor cell proliferation, migration, and colony-forming ability. Transcriptome sequencing integrated with TISIDB (Tumor-immune System Interactions Database) and GEPIA (Gene Expression Profiling Interactive Analysis) databases was performed to identify key cytokines for further functional validation.

**Results:**

In the mouse orthotopic lung cancer model, PM2.5 exposure significantly promoted tumor growth and increased the proportion of M2-type TAMs (*P*<0.05). Subcutaneous co-inoculation with PM2.5-treated BMDMs markedly enhanced tumor proliferation and elevated the intratumoral M2-type TAMs. PM2.5-pretreated BMDMs exhibited an immunosuppressive programmed cell death ligand 1 (PD-L1)^+^/arginase 1 (Arg1)^+^ phenotype, and their conditioned media significantly promoted proliferation, migration, and colony formation of Lewis lung carcinoma cells (LLC) and B16 melanoma cells (B16) (*P*<0.05). Transcriptome analysis revealed that PM2.5 substantially altered macrophage gene expression, with IL-1α identified as a key upregulated secreted cytokine enriched in immunosuppressive related signaling pathways. Clinical database analyses further indicated that IL-1α expression was positively correlated with macrophage and regulatory T cells (Treg) infiltration in the LUAD immune microenvironment, and that high IL-1α expression was associated with worse overall survival in LUAD patients (HR=1.5, *P*=0.0053). Western blot, RT-qPCR, and immunofluorescence confirmed that PM2.5 exposure significantly upregulated IL-1α expression and secretion in TAMs.

**Conclusion:**

PM2.5 exposure facilitates LUAD progression by inducing an immunosuppressive phenotype in macrophages and enhancing the malignant behaviors of tumor cells. Mechanistically, IL-1α may serve as a key pro-tumorigenic cytokine secreted by macrophages under PM2.5 exposure. This study provides new insights into the pathogenesis of PM2.5-associated LUAD and suggests that IL-1α could serve as a potential therapeutic target.

2022年全球癌症统计数据库（GLOBOCAN）的数据^[1]^显示，肺癌仍是全球范围内发病率和死亡率最高的恶性肿瘤之一，2022年全球新发肺癌病例约250万例，死亡人数超过180万例，分别占所有癌症新发病例和死亡病例的12.4%和18.7%。肺腺癌（lung adenocarcinoma, LUAD）是肺癌中占比最高的组织学类型^[2]^，预后普遍不佳（非I期患者5年生存率低于12%-15%）且术后治疗失败率高（约30%-55%，局灶复发和远处转移），亟需从多层面加强其防控策略^[3]^。肿瘤免疫微环境在驱动肿瘤细胞免疫逃逸、促进血管生成和支持转移播散等过程中发挥核心作用，其动态演变直接影响患者对放化疗及免疫治疗的敏感性；因此，靶向重塑或调控免疫微环境被认为是改善LUAD治疗反应和延长生存的重要突破口。

空气污染，尤其是可吸入颗粒物PM2.5的暴露，已被国际癌症研究机构（International Agency for Research on Cancer, IARC）确立为人类一级致癌物。基于流行病学证据分析^[4]^表明，PM2.5暴露与LUAD发生显著相关，其合并风险比（risk ratio, RR）为1.40（95%CI: 1.07-1.83）。PM2.5因其粒径小、比表面积大、易于吸附有毒物质（如重金属、多环芳烃、硫酸盐、硝酸盐等）的特性^[5]^，能够通过呼吸作用进入下呼吸道及肺泡，甚至穿透气血屏障进入全身循环系统。其在肺内沉积后可引发强烈的氧化应激反应与慢性炎症状态，激活多种致癌信号通路，如核因子-κB（nuclear factor kappa-B, NF-κB）信号通路和丝裂原活化蛋白激酶（mitogen-activated protein kinase, MAPK）信号通路，导致DNA损伤、细胞异常增殖及恶性转化^[6]^。因此，深入揭示PM2.5促进LUAD发生发展的免疫与分子机制，不仅有助于理解环境致癌的生物学过程，也为LUAD的早期预防和靶向干预提供了重要的理论依据和潜在的防控策略。

在PM2.5触发肺部慢性炎症与恶性转化过程中，肿瘤免疫微环境的调控起关键作用。肿瘤相关巨噬细胞（tumor-associated macrophages, TAMs）作为肿瘤微环境中最丰富的免疫细胞群体之一，在这一过程中尤为关键。在LUAD中，TAMs通过分泌白细胞介素-6（interleukin-6, IL-6），激活Janus激酶2（Janus kinase 2, JAK2）/信号转导与转录激活因子3（signal transducer and activator of transcription 3, STAT3）/CAAT-增强子结合蛋白β（CAAT-enhancer-binding protein β, C/EBPβ）正反馈通路，从而诱导上皮-间质转化（epithelial-mesenchymal transition, EMT）与肿瘤侵袭及转移^[7]^；同时，肿瘤间质区CD206⁺ TAMs高浸润与LUAD患者的预后显著相关，提示其参与恶性进展并与不良结局相关^[8]^。值得注意的是，肺内巨噬细胞是PM2.5作用的关键靶细胞。在正常情况下，巨噬细胞可通过分泌肿瘤坏死因子-α（tumor necrosis factor-α, TNF-α）、白细胞介素-12（interleukin-12, IL-12）等炎症因子介导抗肿瘤免疫应答^[9]^。然而在PM2.5暴露下，巨噬细胞往往向免疫抑制表型转化，从而促进肿瘤生长、转移与免疫逃逸。其主要机制包括分泌白细胞介素-10（interleukin-10, IL-10）、转化生长因子-β（transforming growth factor-β, TGF-β）等抑制性细胞因子，以及上调程序性细胞死亡配体1（programmed death ligand 1, PD-L1）等免疫检查点分子，进而抑制效应T细胞的功能^[10]^。然而，目前关于PM2.5诱导巨噬细胞免疫抑制性表型的分子机制研究仍较为有限，尤其是关键细胞因子及相应信号通路机制尚未明确。

白细胞介素-1α（interleukin-1α, IL-1α）作为一种重要的早期炎症因子，在肿瘤微环境的形成与调控中具有关键作用。在肺癌研究中，有报道^[11,12]^显示高度转移性LUAD细胞系呈现IL-1α高表达，其异常升高与非小细胞肺癌患者的恶病质密切相关，提示IL-1α可能是影响LUAD患者临床结局的潜在标志物。此外，Kuroda等^[13]^的研究证实，吸入性细颗粒物能够刺激肺泡巨噬细胞产生并释放IL-1α，提示空气污染可能通过免疫细胞介导的途径影响肿瘤发生发展。多项研究^[14,15]^也表明TAMs是肿瘤微环境中IL-1家族细胞因子的一个关键来源，因此，探讨PM2.5是否通过调控IL-1α的表达来影响巨噬细胞极化及LUAD进展，对于深入理解空气污染致癌的免疫机制具有重要意义。

基于此，本研究旨在系统探讨PM2.5暴露是否通过调控巨噬细胞中IL-1α的表达，进而重塑免疫微环境并促进LUAD进展。通过构建体内外PM2.5暴露的小鼠模型，结合转录组测序及生物信息学分析，筛选并验证IL-1α这一核心分子。本研究不仅为PM2.5相关LUAD的机制研究提供了新的视角，也为未来LUAD的免疫治疗策略提供了潜在靶点。

## 1 材料与方法

### 1.1 PM2.5收集及制备

2024年1月至2025年5月在成都市第三人民医院第十层楼露天平台利用空气采样器采集PM2.5。该采样区域位于成都市中心区域，具有人口密集、交通拥堵等特点，能够较为典型地反映成都市城区大气PM2.5的特征。空气采样器每日采集23 h，颗粒物吸附于石英纤维滤膜上。滤膜切成1 cm²小块后浸入无菌水，经超声处理3次提取PM2.5水溶性成分。冷冻干燥后重悬于PBS溶液中，-80 ^o^C保存。使用前再经30 min超声处理以避免PM2.5的聚集。

### 1.2 细胞培养

本实验使用的细胞系包括小鼠Lewis肺癌细胞（Lewis lung carcinoma, LLC）和黑色素瘤细胞（B16 melanoma cells, B16），均购自武汉普诺赛生命科技有限公司；稳定表达荧光素酶的Lewis肺癌细胞（Lewis lung carcinoma cells expressing luciferase, LLC-LUC）购自源井生物。所有实验用的细胞系经STR鉴定正确、支原体检测合格。LLC和LLC-LUC在含10%胎牛血清（fetal bovine serum, FBS）（Gibco, 1932594C）和1%青霉素-链霉素混合液（Gibco, SV30010）的DMEM完全培养基（Gibco, C11995500BT）中培养，B16在含10% FBS和1%青霉素-链霉素混合液的RPMI-1640培养基（Gibco, C22400500BT）中培养，均在37 ^o^C、5% CO₂条件下维持。

骨髓来源巨噬细胞（bone marrow-derived macrophages, BMDMs）的培养参考先前已发表的方法^[16]^。采用无菌PBS多次冲洗小鼠的股骨和胫骨，以收集骨髓细胞悬液。之后，将所得细胞通过70 μm筛网过滤以去除残余组织碎片，并用红细胞裂解液作用约5 min以去除红细胞。处理后的骨髓细胞悬液用含15% FBS及巨噬细胞集落刺激因子（macrophage colony-stimulating factor, M-CSF）（PeproTech, 315-02, 20 ng/mL）的DMEM完全培养基培养7天，并在常规条件下维持，以诱导其分化为巨噬细胞。培养期间每隔2天更换新鲜培养基。

BMDMs细胞完全分化后，将细胞接种于细胞6孔板中，加入LLC细胞培养液上清处理24 h诱导成为TAMs。然后将BMDMs分为PBS组和PM2.5处理组。PM2.5处理浓度为150 μg/mL，处理时间为24 h。处理结束后，分别收集PBS组和PM2.5组BMDMs的细胞培养上清液，按0.22 μm标准过滤后，按上清液与完全培养基体积比1:1混合，制备条件培养基（conditioned medium, CM），分别为PBS-CM和PM2.5-CM。

### 1.3 动物模型的构建

所有动物程序均按照西南交通大学动物保护和使用委员会（SWJTU-2107-004）的指南进行。

#### 1.3.1 原位肺癌模型

构建6-8周龄C57BL/6小鼠适应饲养1周后随机分组。PBS组通过鼻腔滴注PBS溶液（50 μL）；PM2.5组鼻腔滴注PM2.5溶液（50 μL, 5 mg/kg），每日1次，持续1周。然后将处于对数生长期的LLC-LUC细胞与Matrix-Gel^TM^基质胶（Beyotime, C0383）1:1混合，按照0.5×10⁶个细胞/30 μL细胞悬液将LLC细胞接种至小鼠左肺叶，并继续给予PBS或PM2.5鼻腔滴注，隔日1次，持续3周。实验结束后处死小鼠，收集小鼠肺组织。

#### 1.3.2 皮下模型构建

小鼠随机分为PBS组和PM2.5组。使用PBS以及PM2.5（150 μg/mL）处理BMDM细胞后，分别收集BMDM细胞和处于对数生长期的LLC细胞，按照BMDM细胞5×10^6^/mL、LLC细胞1×10^7^/mL的密度制备细胞悬液，然后与Matrix-Gel^TM^基质胶1:1混合。将100 μL细胞混悬液皮下注射于C57BL/6小鼠右侧腋下皮下，1周后观察并测量小鼠肿瘤的大小。待肿瘤大小生长适宜时，收集肿瘤组织，称量肿瘤重量，计算肿瘤体积（V=长×宽²/2）。

### 1.4 小鼠活体成像

小鼠肺原位接种LLC细胞2周后，腹腔注射D-luciferin并使用IVIS Spectrum系统进行成像，成像时间统一设定为注射后10 min，并通过感兴趣区（region of interest, ROI）工具定量光子通量（Photon flux）以反映肿瘤体积。

### 1.5 流式细胞术

使用MA900多应用细胞分选仪（Sony，日本）进行流式检测。分离小鼠肿瘤组织后制备单细胞悬液。细胞经Fc受体封闭（BioLegend, anti-CD16/32）后，于4 ^o^C避光与抗小鼠F4/80-PE抗体（BioLegend, 111604）、抗小鼠CD86-APC抗体（BioLegend, 105012）、抗小鼠CD206（MMR）-Alexa Fluor® 700抗体（BioLegend, 141734）等抗体孵育30 min。洗涤后重悬于PBS溶液进行检测。

BMDMs经PBS和PM2.5处理24 h后，收集细胞，Fc受体封闭后，于4 ^o^C避光条件下与抗小鼠CD274（B7-H1, PD-L1）-PE抗体（BioLegend, 124308）孵育30 min，洗涤后重悬于PBS溶液中进行检测。实验均设置单色补偿及同型对照。实验使用MA900流式细胞分析仪进行检测（Sony，日本），Flow Jo软件进行分析处理。

### 1.6 苏木精-伊红（hematoxylin-eosin, HE）染色

使用4%多聚甲醛溶液固定切除的肿瘤组织24 h，随后对组织进行脱水并进行石蜡包埋，切制厚度为5 μm的连续切片。再经脱蜡、水化、清洗后，进行HE染色，使用200×光学显微镜（IX73，奥林巴斯，日本）进行图像采集，采用CellSens Application Suite软件进行图像分析。

### 1.7 细胞增殖试验（cell counting kit-8, CCK-8）检测细胞增殖能力

取对数生长期的LLC和B16细胞，按5×10^4^个/孔的细胞密度接种于96孔板。根据前述CM分组，每组设置10个复孔，分别以PBS-CM和PM2.5-CM培养细胞24 h，随后每孔加入10 μL CCK-8溶液，1 h后使用酶标仪测定450 nm波长吸光度（optical density, OD）值。CCK-8细胞活性采用以下公式计算：细胞活性（%）=（OD450_PM2.5_−OD450_空白_）/（OD450_PBS_−OD450_空白_）×100%。其中，OD_空白_为空白孔（培养基+CCK-8）。

### 1.8 酶联免疫吸附测定（enzyme-linked immunosorbent assay, ELISA）

利用ELISA方法测定细胞培养上清液中IL-1α的含量。按照试剂盒说明书操作（BioLegend, #433404），使用酶标仪测定450 nm波长的OD值，绘制标准曲线并计算样本中指标的含量。

### 1.9 细胞划痕实验

将处于对数生长期的B16或LLC细胞接种于6孔板中，当细胞生长至约90%融合时，使用100 μL移液枪头垂直划线以形成划痕。用PBS洗涤孔板中漂浮的细胞后，将培养基更换为PBS-CM或PM2.5-CM，分别在0和24 h时使用显微镜拍照，Image J软件统计划痕面积。划痕愈合率按以下公式计算：划痕愈合率（%）=（D_0 h_−D_24 h_）/D_0 h_×100%。其中，D_0 h_为初始细胞间距离均值，D_24 h_为24 h细胞间距离均值。

### 1.10 细胞克隆形成实验

取对数生长期的B16/LLC细胞，以1×10^3^个/孔的密度接种于6孔板，贴壁后分别采用PBS-CM或PM2.5-CM培养，每3天更换培养基，培养2周后使用冰甲醇室温固定30 min，PBS洗涤3次后使用结晶紫避光染色20 min，PBS洗涤3次，晾干后于倒置显微镜下拍照；使用Image J软件对细胞克隆形成的面积进行统计分析。

### 1.11 Western blot

使用RIPA裂解液（Beyotime, P0013B）提取细胞总蛋白，采用BCA试剂盒（Sola rbio, PC0020）检测蛋白浓度。12.5% SDS-PAGE凝胶（Vazyme, E303-01）电泳分离蛋白样品后，恒流将蛋白转至PVDF膜（Merck millipore, ISEQ00010, 0.20 μm），TBST洗膜，5%脱脂奶粉常温封闭1 h，4 °C过夜孵育一抗PD-L1（Cell Signaling Technology, 13684, 1:1000）、IL-1α（Proteintech, 83644-1-RR, 1:2000），室温孵育山羊抗兔IgG二抗（CST, 7074, 1:2000）2 h，使用超敏ECL发光液（NCM, P10300）进行显影，E-BLOT 接触式化学发光成像系统进行图像获取。

### 1.12 逆转录定量聚合酶链反应（reverse transcription quantitative polymerase chain reaction, RT-qPCR）

使用TRIzol提取细胞总RNA，NanoDrop测定RNA浓度及纯度。取2 μg总RNA按照逆转录试剂盒说明书（Vazyme, R223-01）反转录为cDNA。然后，采用SYBR溶液（Vazyme, SQ101）进行定量聚合酶链反应，每个样本均设置3个复孔，以*GAPDH*作为内参基因，采用2^^-ΔΔCt^方法计算相对基因相对表达量。

### 1.13 转录组测序

转录组文库使用TruSeq^TM^ RNA样品制备试剂盒（Illumina, San Diego, CA）构建。文库片段大小通过2%低浓度超琼脂糖凝胶进行检测，并使用Phusion DNA聚合酶进行15个循环的PCR扩增。文库测序由Majorbio公司在Illumina HiSeq Xten平台上采用双端测序（paired-end sequencing）完成。未处理的双端测序读数首先通过SeqPrep和Sickle软件进行去除接头、质量修剪及评估，参数采用默认设置。随后，将质控后的清洗序列（clean reads）利用TopHat软件对参考基因组进行比对，并启用方向性模式。通过每千碱基外显子转录本每百万比对读数（FPKM）计算得到每个转录本的表达水平。

### 1.14 差异表达基因获取及富集分析

根据FRKM所得到的转录本表达水平，使用limma R包分析差异表达基因，|log2（foldchange）|>0.5且*P*<0.05被认定为差异表达基因。在完成差异表达基因的筛选后，进一步进行基因本体论（Gene Ontology, GO）富集分析和京都基因与基因组百科全书（Kyoto Encyclopedia of Genes and Genomes, KEGG）富集分析。GO、KEGG和HALLMARK基因组的gmt文件从MSigDB（http://www.gsea-msigdb.org）下载。使用clusterProfiler R包进行富集分析。统计学显著性定义为*P*<0.05。

可视化热图由https://www.bioinformatics.com.cn/绘制。

### 1.15 在线数据库分析

使用GEPIA（Gene Expression Profiling Interactive Analysis）在线分析工具（http://gepia.cancer-pku.cn/），分析LUAD中IL-1α的表达与预后关系，阈值为**P*<*0.05。通过TISIDB数据库（Tumor-immune System Interactions Database）（http://cis.hku.hk/TISIDB/）分析*IL-1α*基因在LUAD与肺鳞癌（lung squamous cell carcinoma, LUSC）中与肿瘤免疫微环境的关系。选择调节性T淋巴细胞（regulatory T cells, Treg）及巨噬细胞作为研究对象，提取IL-1α表达水平与上述免疫细胞浸润丰度之间的相关性数据，并采用*Spearman*相关性检验计算相关系数（rho）及显著性水平。结果以散点图展示基因表达与免疫细胞丰度之间的相关性。

### 1.16 免疫荧光染色

BMDMs接种于96孔共聚焦培养皿，培养7天后弃去培养基，PBS洗涤后于4%多聚甲醛固定液室温固定30 min，0.1% Triton X-100破膜15 min，10%山羊血清封闭30 min，加入IL-1α抗体4 °C孵育过夜。次日加入Alexa Fluor 647（Abcam, AB150079）标记的山羊抗兔IgG H&L二抗，于室温孵育1 h，使用DAPI染色液进行细胞核复染15 min，并以含50%甘油的封片液封固。

肺组织石蜡切片经脱蜡、水合及抗原修复后，同样以0.1% Triton X-100破膜15 min，10%山羊血清封闭30 min，加入F4/80抗体（Abcam, AB300421）于4 °C孵育过夜，次日加入Alexa Fluor 555（Abcam, AB150078）标记的山羊抗兔IgG H&L二抗，室温孵育1 h，PBS洗涤3次后与IL-1α抗体4 °C孵育过夜，随后加入Alexa Fluor 647标记的山羊抗兔IgG H&L二抗，于室温孵育1 h。最后使用DAPI染色液进行细胞核复染15 min，并以50%甘油封片。使用OLYMPUS共聚焦显微镜（CSU-W1，日本）成像，图像分析采用CellSens Application Suite软件。

### 1.17 统计学方法

所有实验的计量结果以均数±标准误（Mean±SEM）表示，每组实验均独立重复不少于3次。数据分析使用GraphPad Prism 9.0软件完成。两组间比较采用独立样本*t*检验，肿瘤生长曲线采用双因素方差分析进行比较。当*P*<0.05时，认为差异具有统计学意义；所有统计检验均采用双尾分析。

## 2 结果

### 2.1 PM2.5暴露促进小鼠原位肺癌模型中肿瘤的生长

为评估PM2.5暴露对肺部肿瘤进展的影响，我们建立了小鼠原位肺癌模型。在LLC细胞接种前1周对小鼠进行PM2.5预暴露，接种后进行PM2.5隔天暴露（图1A）。小鼠活体成像提示PM2.5组小鼠肺部荧光强度高于PBS组（图1B）；取出肺部肿瘤标本，结果表明PM2.5暴露明显促进了肺部肿瘤的生长（*P*=0.0412，图1C、1D）。HE染色结果提示，PM2.5组癌旁组织中炎性细胞浸润更为明显，且肿瘤组织中血供更丰富（图1E）。为探究PM2.5暴露对TAMs的影响，我们对肿瘤组织及癌旁组织进行流式细胞术分析。结果显示，PBS组与PM2.5组的肿瘤组织和癌旁组织中F4/80⁺ CD86⁺的比例未发现显著差异（*P*=0.2287，*P*=0.7542，图1F、1G）；而PM2.5组中F4/80^+^ CD206⁺细胞群比例在肿瘤组织和癌旁组织中均显著高于PBS组（*P*=0.0190，*P*=0.0004，图1H、1I）。综上，PM2.5促进小鼠原位肺癌的进展，且该过程可能与CD206⁺巨噬细胞（M2型巨噬细胞）比例的上调有关。

**图1:**
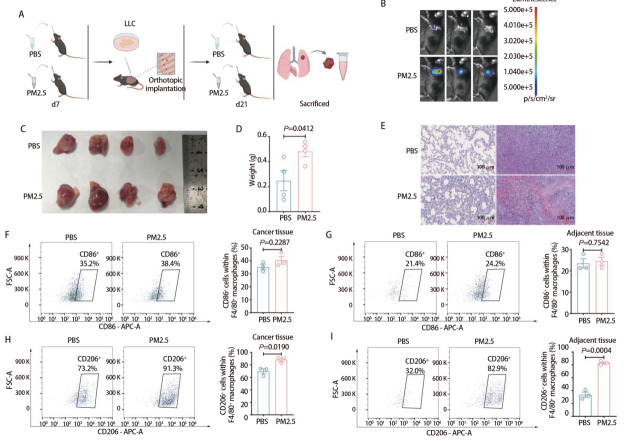
PM2.5暴露促进小鼠原位肺癌模型中肿瘤的生长。A：实验流程：C57BL/6小鼠经鼻腔滴注PBS或PM2.5（5 mg/kg）1周后接种LLC-LUC细胞建立原位肺癌模型，按隔日一次的频率继续鼻腔滴注PM2.5 3周；B：小鼠体内肿瘤活体成像结果；C、D：肺部肿瘤大小对比及肿瘤重量的统计分析；E：原位肺癌组织及癌旁组织HE染色结果展示（比例尺：100 μm）；F-G：肿瘤及癌旁组织中CD86⁺细胞占F4/80^+^巨噬细胞比例的流式分析；H、I：肿瘤及癌旁组织中CD206⁺占F4/80^+^巨噬细胞比例的流式分析。

### 2.2 PM2.5通过诱导巨噬细胞M2型极化促进肿瘤的生长

为进一步验证TAMs在PM2.5暴露促进LUAD中的作用，我们使用PM2.5处理BMDMs细胞后，与LLC细胞共同接种至小鼠皮下（图2A）。结果显示，与PBS组相比，PM2.5组肿瘤体积增长更加快速（图2B），且肿瘤重量明显大于PBS组（*P*=0.0081，图2C、2D），提示PM2.5处理的BMDMs细胞能够促进肿瘤的生长。流式分析显示，两组肿瘤组织中F4/80^+ ^CD86⁺细胞群比例未发现明显差异（*P*=0.0766，图2E、2F），而F4/80^+ ^CD206⁺巨噬细胞在PM2.5组显著增加（*P*=0.0040，图2G、2H）。这些结果提示，PM2.5处理可诱导巨噬细胞向M2型极化，从而促进肿瘤生长。

**图2:**
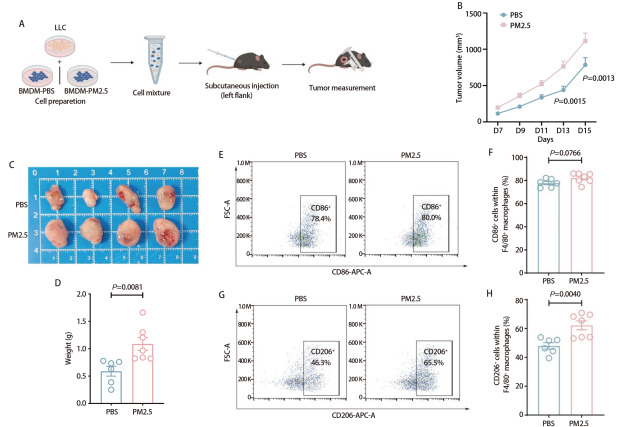
TAMs介导PM2.5暴露促进小鼠皮下移植瘤生长。A：实验流程：BMDMs分别在含PM2.5（150 μg/mL）的培养基或常规处理24 h后，与LLC细胞混合（5×10^6^/mL:1×10^7^/mL）皮下接种；B：PBS组和PM2.5组小鼠皮下肿瘤生长曲线；C、D：皮下接种15天后，取出两组皮下肿瘤，统计肿瘤重量；E、F：肿瘤组织中CD86⁺细胞占F4/80^+^巨噬细胞的流式点图及统计结果；G、H：肿瘤组织中CD206⁺细胞占F4/80^+^巨噬细胞的流式点图及统计结果。

为进一步从体外水平评估PM2.5处理后的巨噬细胞对肿瘤细胞增殖、克隆形成和迁移能力的影响，我们收集PBS组与PM2.5组BMDMs的细胞上清液并制备CM，将PBS-CM与PM2.5-CM分别作用于B16和LLC细胞。CCK-8检测结果显示，B16细胞在PM2.5-CM处理后增殖能力显著增强（*P*<0.0001，图3A），同时划痕实验进一步证实其迁移率显著提高（*P*=0.0010，图3B）。此外，LLC细胞在PM2.5-CM处理后同样表现出更强的增殖和迁移能力（*P*<0.0001，*P*<0.0001，图3C、3D）。克隆形成实验结果发现，PM2.5-CM的处理显著增加了B16和LLC细胞克隆形成的面积（*P*=0.0143，*P*=0.0476，图3E、3F）。上述体内外实验结果进一步证实了PM2.5处理后的巨噬细胞可以明显促进肿瘤细胞的增殖、迁移和克隆形成能力。

**图3:**
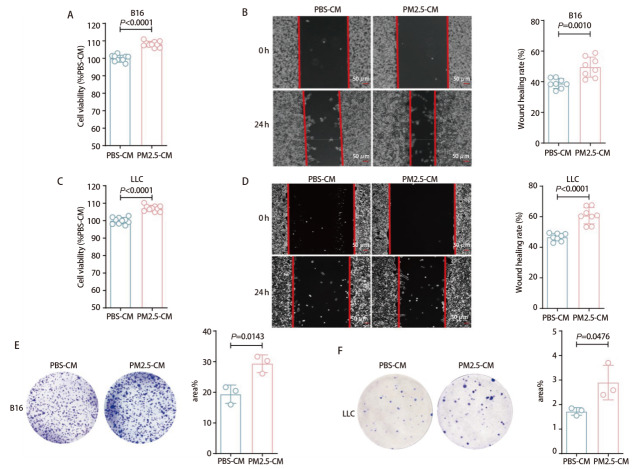
PM2.5-CM促进肿瘤细胞的增殖、迁移和克隆形成。A：CCK-8检测B16细胞增殖能力；B：划痕实验评估B16细胞迁移能力（比例尺：50 μm）；C：CCK-8检测LLC细胞增殖能力；D：划痕实验评估LLC细胞迁移能力（比例尺：50 μm）；E、F：克隆形成实验检测B16与LLC细胞的克隆形成能力。

### 2.3 PM2.5诱导巨噬细胞免疫抑制表型的改变

为揭示PM2.5对巨噬细胞表型的影响，我们检测了巨噬细胞M2标志物精氨酸酶1（arginase 1, Arg1）和免疫检查点分子程序性细胞死亡配体1（programmed cell death ligand 1, PD-L1）的表达。RT-qPCR结果显示，与PBS组相比，PM2.5组巨噬细胞中Arg1表达显著上调（*P*=0.0025，图4A）。同时，Western blot结果显示PM2.5暴露能够明显促进巨噬细胞PD-L1蛋白水平的表达（*P*=0.0014，图4B、4C）。流式细胞术进一步证实F4/80^+ ^PD-L1⁺细胞比例显著升高（*P*<0.0001，图4D、4E）。上述结果提示，PM2.5诱导巨噬细胞获得免疫抑制/促肿瘤相关表型（PD-L1^+^、Arg1^+^），为其促进肿瘤细胞增殖与迁移提供了分子层面的依据。

**图4:**
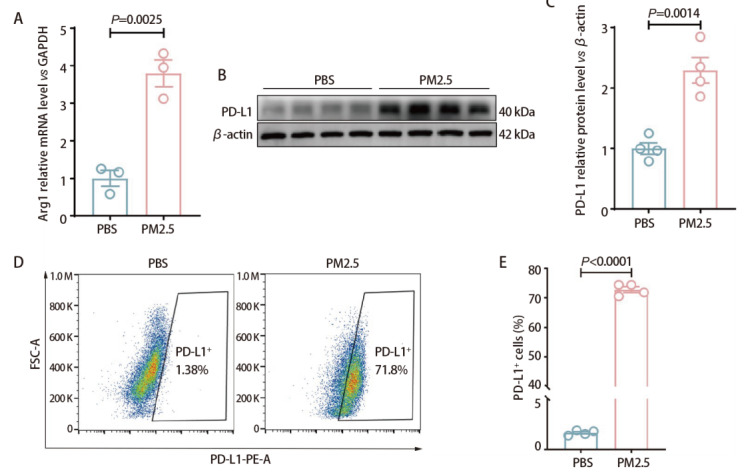
PM2.5暴露诱导TAMs获得免疫抑制表型。A：RT-qPCR检测BMDMs细胞中Arg1的mRNA表达（GAPDH归一化）；B、C：Western blot检测BMDMs细胞PD-L1蛋白表达（β-actin归一化）；D、E：流式分析检测PD-L1^+ ^BMDMs细胞群。

### 2.4 转录组学分析提示IL-1α参与PM2.5诱导的巨噬细胞促癌作用

为进一步探究PM2.5诱导的巨噬细胞表型变化的机制，我们对PM2.5处理后的BMDMs进行了转录组测序。基因本体GO分析显示，与PBS组相比，PM2.5组上调差异基因的分子功能（biological process, BP）显著富集于炎症反应和免疫反应（图5A）；细胞组分（cellular component, CC）主要分布于细胞外基质、细胞质及细胞膜等区域；分子功能（molecular function, MF）则涉及蛋白质结合、同源蛋白结合及蛋白质同源二聚化活性等（图5A）。KEGG分析提示上调差异基因涉及细胞因子-受体相互作用、趋化因子信号通路、MAPK通路等（图5B）。值得注意的是，这些富集条目中有多项与肿瘤发生发展密切相关，例如炎症反应和免疫反应可驱动免疫抑制性微环境的形成，细胞因子-受体相互作用及趋化因子信号则直接参与肿瘤细胞的增殖、迁移与免疫逃逸，而MAPK通路作为经典的促癌信号轴同样被显著激活。鉴于PM2.5-CM对肿瘤细胞增殖的显著促进作用，我们推测BMDMs对癌细胞的影响可能依赖其分泌的某些细胞因子驱动。基于此，我们进一步聚焦于这些促癌相关条目，并通过交集分析筛选出3个关键上调基因（*IL-1α*、*PPBP*、*LAT*），它们同时富集于免疫反应和炎症反应两个与免疫抑制性微环境密切相关的条目中。在3个交集基因中，*IL-1α*作为分泌型炎症因子，与免疫抑制和信号通路激活密切相关，因此被选为候选基因（图5C）。基于癌症基因组图谱（The Cancer Genome Atlas, TCGA）的公开转录组测序数据，我们进一步利用TISIDB数据库分析IL-1α表达与免疫细胞浸润的关系。结果（图5D）显示，在LUAD（*n*=517）中，IL-1α的表达水平与巨噬细胞和Treg细胞的浸润丰度均呈显著正相关（分别为rho=0.437和0.466，*P*<0.0001）；在LUSC（*n*=501）中，IL-1α与巨噬细胞浸润无显著相关性（rho=0.069, *P*=0.1220），但与Treg浸润相关（rho=0.137, *P*=0.0021）。这些结果提示，IL-1α可能通过促进免疫抑制性细胞的浸润，在LUAD中参与塑造免疫抑制性微环境。生存分析结果进一步提示，LUAD和LUSC患者中IL-1α的高表达与不良预后显著关联（HR=1.5，*P*=0.0053，图5E）。这些结果提示IL-1α可能参与免疫抑制微环境的形成，并且其高水平表达与LUAD患者不良预后密切关联。

**图5:**
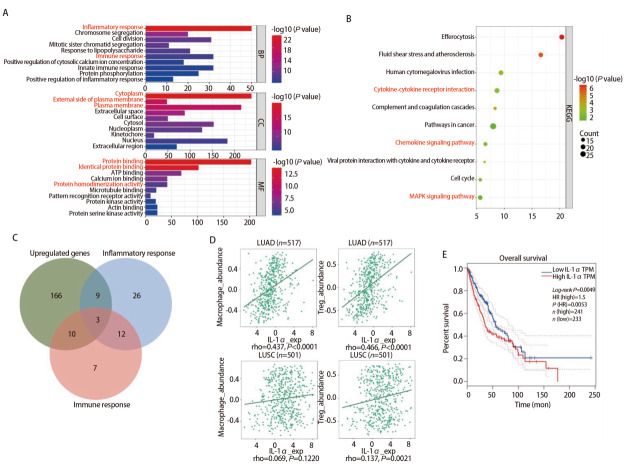
转录组分析显示IL-1α是PM2.5暴露诱导巨噬细胞分泌的关键促癌因子。A：GO富集分析；B：KEGG通路富集分析；C：对免疫相关GO条目（免疫反应和炎症反应）与上调基因交集后得到3个交集基因（IL-1α、PPBP、LAT）；D：TISIDB数据库分析IL-1α在LUSC与LUAD中免疫细胞浸润相关性；E：Kaplan-Meier曲线展示IL-1α表达量与LUAD及LUSC患者预后之间的关系。

### 2.5 PM2.5暴露促进巨噬细胞IL-1α表达量的上调

为验证上述推测，我们首先检测了BMDMs细胞内及其上清液中的IL-1α水平。Western blot结果显示PM2.5暴露能够明显上调BMDMs细胞中IL-1α的表达（*P*=0.0292，图6A、6B）。ELISA定量检测结果提示PM2.5暴露明显促进了巨噬细胞IL-1α的分泌（*P*=0.0059，图6C）。RT-qPCR进一步证实了IL-1α mRNA水平在PM2.5处理后明显上调（*P*=0.0003，图6D）。细胞免疫荧光染色显示，PM2.5组BMDMs细胞中IL-1α荧光强度显著增强（*P*=0.0007，图6E、6F）。此外，在小鼠原位肺癌模型的肿瘤组织石蜡切片中，我们也观察到PM2.5组肿瘤组织内巨噬细胞IL-1α表达量的明显增加（图6G）。以上体内外的结果共同表明，PM2.5暴露能够促进巨噬细胞IL-1α的上调及向细胞外的分泌，提示IL-1α可能是巨噬细胞促进LUAD进展的关键细胞因子。

**图6:**
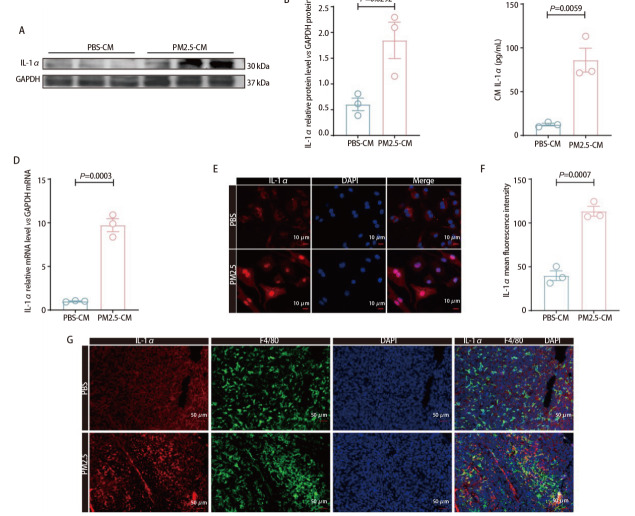
PM2.5暴露上调巨噬细胞IL-1α的表达。A、B：Western blot定量分析BMDMs中IL-1α蛋白水平（GAPDH归一化）；C：ELISA测定BMDMs上清液中IL-1α蛋白水平；D：RT-qPCR检测BMDMs中IL-1α的mRNA表达（GAPDH归一化）；E、F：BMDMs细胞免疫荧光染色（比例尺：10 μm）；G：小鼠原位肺癌组织石蜡切片的免疫荧光染色（比例尺：50 μm）。

## 3 讨论

LUAD是肺癌中最常见的组织学亚型，也是全球范围内癌症相关发病率和死亡率的主要致因之一，空气污染与其发病及进展密切相关。多项队列研究证实，长期接触PM2.5与LUAD的不良预后呈显著相关性，Yang等^[17]^的病例对照研究显示，PM2.5水平每增加10 μg/m³，LUAD患者的发病风险上升约25%（OR=1.25, 95%CI: 1.07-1.46），提示持续高水平PM2.5暴露可能在LUAD的发生及预后不良中均发挥重要作用。既往研究^[18,19]^表明，炎症介质的释放与致癌信号通路的激活是PM2.5促癌的重要机制。此外，PM2.5还可以通过重塑肿瘤免疫微环境加速疾病进展，其中巨噬细胞在这一过程中发挥核心调控作用^[10]^。本研究系统揭示了PM2.5通过诱导巨噬细胞分泌IL-1α并获得免疫抑制表型，进而促进LUAD进展。

本研究中，小鼠原位肺癌模型结果显示PM2.5处理能够显著增强肺部肿瘤的生长能力，且伴随肿瘤组织血供增加及癌旁组织的炎症反应加重。Chang等^[20]^研究提示空气污染颗粒可通过激活肺泡巨噬细胞缺氧诱导因子-1α（hypoxia-inducible factor-1α, HIF-1α）以加速糖酵解，导致乳酸堆积，并伴随免疫抑制性标志物CD206、PD-L1和PD-L2的上调；此外，Park等^[21]^研究发现颗粒物还能增加巨噬细胞肝素结合表皮生长因子（heparin-binding epidermal growth factor, HBEGF）的分泌，该因子通过激活癌细胞的表皮生长因子受体（epidermal growth factor receptor, EGFR）通路诱导EMT并提高迁移/侵袭能力，促进癌症的转移。本研究的实验结果同样提示，在原位及皮下肿瘤模型中，PM2.5暴露导致M2型巨噬细胞比例显著上升，并伴随肿瘤生长加速。进一步的体外实验亦表明，经PM2.5处理的BMDMs呈现PD-L1^+^、Arg1^+^的免疫抑制表型。既往研究显示，TAMs可通过上调免疫检查点分子PD-L1，经由程序性死亡受体1（programmed cell death 1, PD-1）/PD-L1轴直接抑制CD8⁺ T细胞活性^[22]^；同时，其高水平表达的Arg1可耗竭肿瘤微环境中的精氨酸，从代谢层面限制T细胞增殖与效应功能^[23]^。

肿瘤免疫微环境的形成不仅依赖于免疫检查点和代谢通路，还受多种免疫细胞及其分泌因子的协同调控。例如，TAMs分泌的TGF-β和IL-10可抑制CD8⁺ T细胞的增殖、效应功能及杀伤活性，从而削弱抗肿瘤免疫反应^[24]^；与此同时，Tregs通过分泌IL-35等抑制性因子，不仅能促进其他免疫抑制细胞的募集，还能增强免疫逃逸机制^[25]^。本研究中，PM2.5-CM可显著促进肿瘤细胞的增殖、迁移和克隆形成能力，提示PM2.5处理后的巨噬细胞可通过其分泌因子重塑肿瘤微环境，从而发挥促瘤作用。转录组学分析结果进一步揭示了PM2.5处理后巨噬细胞的免疫调节特征：上调差异基因在炎症反应、免疫应答等GO条目，以及细胞因子-受体相互作用、趋化因子信号通路、MAPK信号通路等KEGG条目中显著富集。根据交集分析，我们筛选IL-1α作为关键促癌细胞因子。

IL-1α作为IL-1家族的重要成员，既可作为细胞内预警分子，也能通过IL-1R1激活NF-κB/MAPK，上调基质金属蛋白酶（matrix metalloproteinases, MMPs）、诱导血管生成并驱动免疫抑制，进而调节炎症并促进肿瘤生长^[26-28]^。此外，有研究^[29]^提示PM2.5可通过核苷酸结合寡聚结构样蛋白3（NLR family pyrindomain containing 3, NLRP3）炎症小体促使巨噬细胞分泌IL-1β，这表明IL-1家族成员在颗粒物诱导的免疫反应中可被激活，为我们聚焦于IL-1α提供依据。通过RT-qPCR、ELISA、Western blot及免疫荧光的结果，我们证实IL-1α在PM2.5组巨噬细胞及肿瘤组织中均显著上调。临床数据库的相关性分析进一步支持了这一结论：在LUAD及LUSC患者中，IL-1α的高表达与Treg和巨噬细胞浸润呈正相关，并与总体生存率下降密切相关。这些结果与既往关于IL-1α促癌作用的报道相呼应：既有研究^[27]^表明IL-1α可通过重塑肿瘤免疫微环境促进肿瘤进展与转移，亦有研究^[30]^揭示其在驱动肿瘤对靶向治疗耐受中发挥重要作用；此外，临床循证资料^[31]^提示IL-1α的高表达与胰腺癌患者不良预后密切相关。结合本研究结果，提示IL-1α可能是PM2.5诱导LUAD免疫抑制性微环境形成的关键分子。值得注意的是，多项体外/体内研究^[32,33]^表明PM2.5可激活巨噬细胞中的NF-κB与MAPK（p38/ERK）信号，而IL-1家族因子亦被证明能够通过这些通路驱动炎症反应和肿瘤进展。与本研究的转录组学提示MAPK信号通路显著激活的结论一致，进一步增强了IL-1α作为核心分子的生物学合理性。

综上所述，本研究揭示了PM2.5通过诱导巨噬细胞免疫抑制性转化及IL-1α上调，促进LUAD进展的潜在机制。IL-1α有望成为PM2.5相关LUAD的治疗干预靶点，未来研究可聚焦于评估IL-1α阻断是否能够逆转PM2.5诱导的免疫抑制微环境及促癌效应，从而为环境相关肿瘤的防治提供新的理论依据与干预策略。
